# Exploring the Interplay between Carotenoids, Lipid Oxidation, and Cognitive Impairment in Aging

**DOI:** 10.1002/alz.087972

**Published:** 2025-01-09

**Authors:** Irundika HK Dias, Sewa Abdullah, Lena Pickert, Alexander Thimm, Dumindri Abeysena, Joris Deelen, Helen R Griffiths, Maria Cristina Polidori

**Affiliations:** ^1^ Aston University, Birmingham, West Midlands United Kingdom; ^2^ Cologne University Hospital, Cologne, Cologne Germany; ^3^ Max‐Planck‐Institute for Biology of Ageing, Cologne, Cologne Germany; ^4^ University of Swansea, Swansea, swansea United Kingdom; ^5^ University Hospital of Cologne, Köln, Cologne Germany

## Abstract

**Background:**

Alzheimer's disease (AD) is a progressive neurodegenerative disorder characterised by cognitive decline, memory loss, and impaired daily functioning. As the global population ages, the prevalence of AD continues to rise, emphasising the urgent need for effective preventive and therapeutic strategies. Carotenoids, a group of naturally occurring pigments with antioxidant properties, have gained attention for their potential neuroprotective effects. Carotenoids interrupt the oxidative process by scavenging free radicals and quenching singlet oxygen, ultimately reducing lipid oxidation and preserving the integrity of lipids in biological systems. This project investigated the relationship between blood carotenoid levels, lipid oxidation and their impact on cognitive impairment.

**Method:**

Serum samples were collected from 49 healthy individuals (17 male, 32 female) who are 65 years or older. Serum carotenoids (Retinol, Lutein, Zeaxanthin, Lycopene, Alpha‐Tocopherol, β‐Carotene) were analysed by reversed phase HPLC method using methanol/acetonitrile/water gradient. Method validation was performed to establish linearity, sensitivity, recovery, and accuracy. Lipid oxidation products (oxysterols and oxidised phospholipids) were analysed using targeted mass spectrometry methods developed in our laboratory. Cognitive functions were measured using Montreal Cognitive Assessment, Trail Making Tests, letter fluency, category fluency and CogState.

**Result:**

HPLC assay for carotenoids assay showed a linear dynamic range (R2 > 0.94) of 0.01 µM‐20 µM for the six carotenoids that were tested. High intra‐ and inter‐day precision (CV < 9%) and high concentration accuracy (< 15% absolute error) were observed for the QC plasma samples. Correlation analyses did not show any relationship between cognitive status with serum carotenoids or oxysterols; 7‐ketocholesterol, 7β hydroxycholesterol and 25‐hydroxycholesterol and 27‐hydroxycholesterol). 27‐hydroxycholesterol was increased in individuals >80 years old.

**Conclusion:**

Our data suggests some lipid oxidation products such as 27‐ hydroxycholesterol may increase with age. However, further analysis is needed to conclude how lipophilic carotenoids may associate with lipid oxidation and cognitive impairment.

